# PW02-023 - Qualitative aspects of autoinflammatory diseases

**DOI:** 10.1186/1546-0096-11-S1-A163

**Published:** 2013-11-08

**Authors:** D Konukbay, D Yildiz, C Acikel, D Karaman, B Eren-Fidanci, Y Bilginer, I Koné-Paut, J Frenkel, M Gattorno, S Ozen, E Demirkaya

**Affiliations:** 1 Pediatric Rheumatology, Gulhane Military Medical Academy, School of Nursing, Ankara, Turkey; 2 FMF Arthritis Vasculitis and Orphan Disease Research Center, Ankara, Turkey; 3 Child and Adolescent Psychiatry, Gulhane Military Medical Faculty, Ankara, Turkey; 4 Pediatric Rheumatology, Hacettepe University, School of Medicine, Ankara, Turkey; 5 Pediatric Rheumatology, University of Paris SUD, Paris, France; 6 Pediatrics, University of Utrech , Utrech, The Netherlands; 7 Pediatric Rheumatology, Ospedale Gaslini, Genoa, Italy; 8 Pediatric Rheumatology, Gulhane Military Medical Faculty, Ankara, Turkey

## Introduction

In pediatric rheumatology, the lack of scales showing activities of illness in the patients groups, the absence of biomarkers for the severity of damage led the scientific world to develop a scale where the patient can make an self-assessment with quantitative results. So, a necessity has been occurred to develop a multidimensional scale which is understandable, applicable and comprehensive in the evaluation of children with auto-inflammatory diseases.

## Objectives

The aim of this study is to develop a multidimensional assessment instrument named "**J**uvenile **A**uto**i**nflammatory Disease **M**ultidimensional **A**ssessment **R**eport" (JAIMAR) to measure all the domains of the autoinflammatory diseases. In this study the data of "Qualitative Interviews", one of the steps of item generation in JAIMAR, will be presented.

## Methods

19 mothers who have children with autoinflammatory disease (8 FMF, 5 Behcet, 4 PFAPA, 1 HIDS, 1 TRAPS) and their children greater than 7 years old were enrolled in this study. Data were collected using both a demographic data form and a semi-structured interview form. The study was performed on individual patient face-to face interview. Data were collected by using both a demographic data form and a semi-structured interview form. Data analysis by grounded theory and N Vivo 10 software.

## Results

Unknowing the time of attack, lifelong illness, difficulties in diagnosis and exposure to the other parts of the body were described as the worst parts of the illness. In addition to physical factors such as cold and fatigue, psychological factors such as overexcitement, worry and happiness were stated to be in the triggering factors of the attacks. Although decrease in attacks after treatments were stated, lifelong drug addiction and its side effects were told to be the most worrying aspects. Problems at school (absenteeism, loss of performance, fear of having attack at school and bad peer relations) were explained as the biggest difficulties affecting the quality of life. Problems with friends, precocity, and extreme expressions such as depression/wanting to die due to back pain were to be the in the emotional difficulties.

## Conclusion

These results provide an evidence based data for the assessment of children with autoinflammatory disease by several domains including physical, emotional and social aspects as well as treatment protocols. With this regard there is a need to develop a multidimensional instrument to measure important aspects of the illness gained from these results.

## Disclosure of interest

None declared.

